# Extraocular sebaceous carcinoma of the chest wall: A case report

**DOI:** 10.1016/j.ijscr.2023.108195

**Published:** 2023-04-13

**Authors:** Dakita Mack, Mohamed A. Hussein, Gul Sachwani-Daswani, Kristoffer Wong

**Affiliations:** a Department of General Surgery, Beaumont Health Farmington Hills, Farmington Hills, MI, USA; b Division of Trauma Surgery, Department of General Surgery, Hurley Medical Center, Flint, MI, USA

**Keywords:** Extraocular, Sebaceous carcinoma, Case report, Muir-Torre, Mismatch repair, Lynch syndrome

## Abstract

**Introduction:**

Sebaceous carcinoma is a rare malignancy that is most commonly found in the head and neck region, particularly in the periocular region. Extraocular lesions are rarer, however are thought to have a higher association with Muir-Torre Syndrome, a variant of Lynch Syndrome, that affects both sebaceous glands and visceral organs.

**Case presentation:**

Our patient was a 54 year old male with a past medical history of hypertension, stroke, and vertigo who presented to the emergency department with paresthesias concerning for a transient ischemic attack in the setting of a hypertensive emergency. After admission, the patient reported an abscess on the chest that was present for four months prior. Subsequent physical exam incidentally revealed a fungating mass located on the chest wall. The mass was noted to have central ulceration and necrosis.

**Clinical discussion:**

CT scan of the chest with intravenous contrast revealed that the mass did not invade the chest wall, therefore a surgical excision was performed. A final diagnosis of sebaceous carcinoma was made after microscopic examination of the resected mass. Immunohistochemistry revealed loss of expression of mismatch repair genes. The patient was lost to follow-up before any additional work up could be performed.

**Conclusion:**

Extraocular sebaceous carcinomas should be tested using immunohistochemistry for loss of expression of mismatch repair genes. Patients with loss of expression should be risk stratified using the Mayo Muir-Torre risk score to determine if they should undergo germline genetic testing for Lynch Syndrome. Patients with issues in adherence represent a unique diagnostic challenge in potentially evolving diseases.

## Highlights

• Sebaceous carcinoma is a rare malignancy that is most often found in the periocular region.
• Extraocular manifestations usually have a higher association with Muir-Torre Syndrome.
• The Mayo Muir-Torre risk score should be used to determine need for germline genetic testing for Muir-Torre Syndrome.
• Non-adherence represents a unique diagnostic challenge in potentially evolving disease.

## 1 Introduction

Sebaceous carcinoma is a rare, but potentially aggressive malignancy. Given their sebaceous gland origin, they may arise anywhere these glands are located. The periocular variant, arising from the ocular adnexa, is by far the most common presentation of sebaceous carcinoma. However, sebaceous carcinoma can uncommonly arise in an extraocular location, the most frequent being the head and neck [1]. A 2011 analysis of SEER data demonstrated that periocular tumors have a higher incidence of metastasis, but there was no statistically significant difference in cause-specific mortality based on sebaceous tumor location [2]. In terms of staging, extraocular variants are staged using the AJCC TNM staging guidelines for non-melanoma skin cancer while the AJCC staging for eyelid carcinomas is used for the periocular variant [3].

Extraocular lesions are thought to have a higher association with Muir-Torre syndrome [4], an autosomal dominant mutation in mismatch repair genes that affects both sebaceous glands and visceral organs [5]. The mainstay of treatment for both variants is surgical excision, with either the more tissue-sparing Mohs micrographic surgery or wide local-excision [3].

Here we present a case of an incidentally discovered cutaneous sebaceous carcinoma that was originally thought to be a chest wall mass of infectious etiology. The clinical course of this case occurred in its entirety at a 457 bed community-based, university-affiliated medical center. This case has been reported in line with the SCARE 2020 guidelines [6].

## 2 Case presentation

Our patient is a 54 year old White/Caucasian male with past medical history of hypertension, stroke, and vertigo who was brought by ambulance to the emergency department with left-sided numbness concerning for a transient ischemic attack in the setting of an hypertensive emergency (SBP > 200). He has never smoked and reports no drug use, but has a history of excessive alcohol use (>20 standard drinks per week). There was no pertinent family history. Following an emergency department workup that consisted of unremarkable blood work, an MRI that revealed a new posterior pontine susceptibility, and a head CT that was negative for new changes, the patient was started on clopidogrel and admitted to the medical ward for control of blood pressure with gradual improvement of symptoms.

**Fig. 1:**
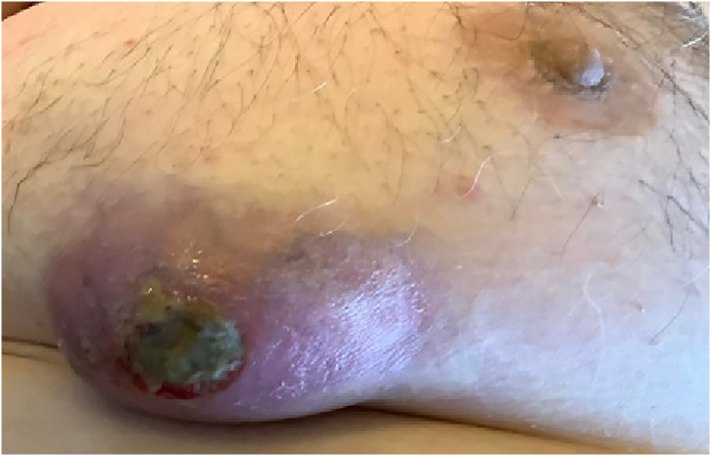
Clinical photograph showing a mass with central ulceration or eschar present on the chest wall in the 8 o'clock position in relation to the patient's right nipple.

**Fig. 2:**
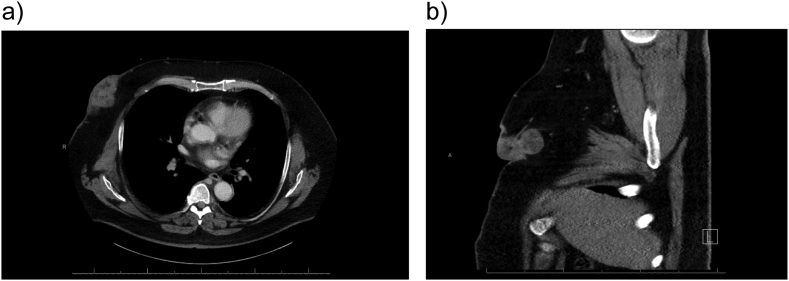
Computed tomography images showing the location and extension of the chest wall mass. a) axial cuts b) sagittal cuts.

During his stay, the patient voiced concerns of an ‘abscess’ of his right chest that had been present for 4 months prior to hospitalization, after which the general surgery service was consulted. A physical examination revealed a painless mass measuring 5 × 6 × 5 cm,with central ulceration and mild spontaneous and intermittent brown-colored discharge (Fig. 1). He firmly denied any accompanying fever/chills, tenderness, nausea/vomiting and malaise. An ultrasound examination revealed a complex mass with peripheral increased vascularity which at the time endorsed an infectious process. A computed tomography of the chest with IV contrast (Fig. 2) revealed a soft tissue mass that did not invade the chest wall. Following the retrieval of aerobic and anaerobic cultures, the patient was started on intravenous clindamycin and medically optimized using hydralazine for surgical excision.

**Fig. 3:**
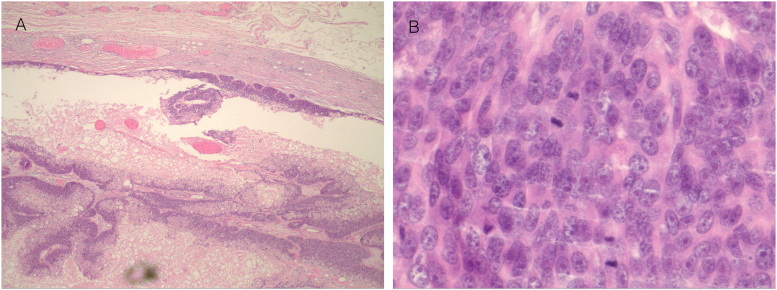
Pathology slides of the tumor with hematoxylin-eosin staining. A) Central large cystic area filled with sebum. (Low power at 2×). B) Basaloid cells showing cytologic atypia and increased mitotic activity. (High power 40×).

The next day the patient was brought to the operating room for the procedure which was performed by a consultant general surgeon with assistance from a resident physician. Following successful excision using a 3:1 transverse elliptical incision, the specimen was sent with inked margins for examination by the pathology department. Sections of the specimen revealed a central well-circumscribed soft yellow lesion measuring 6 × 5 × 3.4 cm. The encapsulated lesion grossly extended to within 1 mm of the inked resection margin and all margins were considered to be free. Fig. 3 shows microscopic views of the resected mass. The mass was later sent for additional pathologic examination and immunohistochemical (IHC) analysis for DNA mismatch repair defects. Table 1 contains a summary of the results of the IHC analysis.

**Table 1:** Results from immunohistochemical staining of the tumor demonstrating loss of nuclear expression of MSH2 and MSH6.

Gene	Expression
MLH1	Intact nuclear expression
MSH2	**Loss** of nuclear expression
MSH6	**Loss** of nuclear expression
PMS2	Intact nuclear expression

Following the procedure, results of previously obtained cultures revealed growth of Methicillin-sensitive *Staphylococcus aureus* after which the patient's antibiotic coverage was switched to intravenous vancomycin. The patient was then discharged the following day on oral cephalexin with recommendations to follow up at the outpatient surgery clinic and to undergo a colonoscopy to exclude any occult gastrointestinal malignancies. Although the patient consented for removal of the mass, he has not followed up with the surgeon after discharge, nor is there any indication that he underwent the recommended colonoscopy. Several attempts have been made to contact the patient for continuity of care and to risk-stratify this patient, however to no avail. A retrospective calculation of the Mayo Muir-Torre score was not possible due to the lack of a documented negative family history, specifically for Lynch-related cancers.

## 3 Discussion

Sebaceous carcinoma (SC) is a rare and aggressive malignancy that is most commonly found in the periocular region; however, it can present anywhere on the body where there are sebaceous glands [1]. Sebaceous carcinomas, like other tumors, have the potential for insidious growth. Wrightson et al. and Hasan et al. have both described incidences of rare tumors growing otherwise undetected until they are discovered underlying a more acute process such as an abscess [7], [8]. Treatment for sebaceous carcinoma is largely based on surgical resection. Given the predominance of ocular region involvement and the cosmetic sensitivity of this area, Mohs micrographic surgery is an option for many patients. In a 2020 review, Wu et al. found that studies reporting treatment outcomes using MMS showed lower rates of local recurrence, nodal metastasis, and death due to disease when compared to excision. However, the majority of these studies report these outcomes in ocular SC rather than extraocular and on average, margins between 3 and 5 mm were used when excision was performed [9].

Extraocular tumors can be treated with Mohs micrographic resection in areas of cosmetic importance; they can also be treated using excision and margin control, albeit with wider margins varying from 5 mm to 20 mm [9]. Recent guidelines suggest, although weakly (GRADE C: 2A), that when Mohs is not available, a wide local excision with 10 mm peripheral margins and excision down to the level of fascia is recommended [3]. Routine use of sentinel lymph node biopsy is not recommended [3]. Pathologically, extraocular sebaceous carcinomas invade the dermis, however rarely extend to subcutaneous tissue and muscle [3]. There is limited data to support adjuvant radiation for these tumors [3].

Periocular tumors differ from extraocular tumors in frequency, metastatic potential, and prognosis. While periocular tumors are known to have a poorer prognosis due to higher metastatic potential [2], extraocular tumors are thought to have a better prognosis but tend to have an increased association with Muir-Torre syndrome [4]. Muir-Torre, a variant of Lynch Syndrome, is an autosomal dominant disease caused by deletion of Mismatch Repair gene (MMR) that causes at least one sebaceous malignancy with another lesion of at least one visceral organ, most commonly colorectal adenocarcinoma; however, additional sites of malignancy can include the small bowel, brain, breast and lung [10]. Immunohistochemistry (IHC) testing of the tumor is often reflexively performed to assess for mismatch repair protein loss. The correlation of positive IHC testing when performed on sebaceous tumors with germline mutation of Muir-Torre or Lynch Syndrome is roughly 81–85 % sensitive and 48 % specific, compared with 92–94 % sensitivity and 88–100 % specificity when IHC is performed on colonic tumors [3].

Given the lower sensitivity and specificity of IHC for sebaceous carcinomas when compared to colorectal tumors, it is debated whether patients with extraocular sebaceous carcinoma should be regularly screened for Muir-Torre using germline genetic testing. The Mayo Muir-Torre risk score was created for guidance [11]. The scoring system is based on four factors: age at diagnosis, total number of sebaceous neoplasms, as well as personal and family history of any Lynch-related cancers. Each factor was given a score of 0 or 1 with the exception of the total number of sebaceous neoplasms (SN); 1 SN was given a score of 0 and 2+ SN was given a score of 2. On a scale of 0–5, patients with a score of 2 or higher should undergo germline genetic testing for loss of MMR protein [11]. Universal Muir-Torre screening as well as screening in periocular lesions is not routinely recommended [3]. Despite a worse prognosis and higher potential for metastasis in periocular tumors, there was no statistically significant difference in survival when comparing pericocular tumors to extraocular tumors [2], [12].

## 4 Conclusion

Sebaceous carcinoma is a rare malignancy that is most often found in the periocular region. It is even more rare to find such a tumor in an extraocular location such as the chest wall. It is common to reflexively perform IHC testing on an extraocular tumor for mismatch repair genes; however, loss of expression (thus a positive test) is not predictive of the presence of Muir-Torre and Lynch Syndrome. Although germline genetic testing is diagnostic, a more focused approach should be taken in deciding which patients to investigate using the Mayo Muir-Torre risk score. Adjunctive screening could be accomplished with colonoscopy. In patients who are known to have challenges with compliance and adherence, it may be beneficial to perform additional testing in the index admission for a complete workup as they represent a unique diagnostic challenge in potentially evolving disease.

## Consent

Written informed consent was obtained from the patient for publication of this case report and accompanying images. A copy of the written consent is available for review by the Editor-in-Chief of this journal on request.

## Ethical approval

Anonymized case reports are exempt from ethical approval and do not require IRB review at our institution.

## Funding

None.

## Author contribution

DM (Conceptualization, Investigation, Writing – Original Draft); MH (Investigation, Writing – Original Draft, Writing – Review and Editing, Data Curation, Visualization); GS (Conceptualization, Writing – Original Draft, Writing – Review and Editing, Supervision); KW (Conceptualization, Investigation, Supervision).

## Guarantor

Kristoffer Wong, DO, FACOS.

## Research registration number

Not applicable.

## Declaration of competing interest

None.
